# *N*-(3-Bromo-1,4-dioxo-1,4-dihydro-2-naphth­yl)-2-chloro-*N*-(2-chloro­benzoyl)benzamide

**DOI:** 10.1107/S1600536808039214

**Published:** 2008-12-06

**Authors:** Emmanuel S. Akinboye, Ray J. Butcher, Yakini Brandy, Tolulope A. Adesiyun, Oladapo Bakare

**Affiliations:** a Department of Chemistry, Howard University, 525 College Street NW, Washington, DC 20059, USA

## Abstract

The title compound, C_24_H_12_BrCl_2_NO_4_, was synthesized from 2-amino-3-bromo-1,4-naphthoquinone and 2-chloro­benzoyl chloride. The crystal structure shows that each of the chloro­phenyl rings is inclined at about 60° to the naphthoquinone ring system. The two chloro­phenyl rings adopt a conformation that ensures that chlorine substituents are *anti* so as to reduce electronic repulsion. An examination of the packing shows close O⋯Br and Cl⋯Cl contacts of 2.947 (2) and 3.346 (1) Å, respectively. In addition, the molecules are linked by weak intermolecular C—H⋯O and C—H⋯Cl interactions.

## Related literature

For similar structures, see: Lien *et al.* (1997 ▶); Huang *et al.* (1998 ▶); Bakare *et al.* (2003 ▶); Copeland *et al.* (2007 ▶); Win *et al.* (2005 ▶); Rubin-Preminger *et al.* (2004 ▶). For the properties of compounds with the chloro-1,4-naphthoquinone skeleton, see: Chang *et al.* (1999 ▶); Ertl *et al.* (1999 ▶).
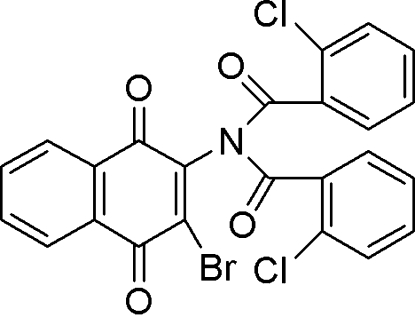


## Experimental

### 

#### Crystal data

C_24_H_12_BrCl_2_NO_4_
*M* *_r_* = 529.16
Monoclinic, 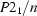

*a* = 12.8590 (3) Å
*b* = 7.81260 (10) Å
*c* = 21.9574 (4) Å
β = 106.272 (2)°
*V* = 2117.53 (7) Å^3^
*Z* = 4
Mo *K*α radiation
μ = 2.23 mm^−1^
*T* = 200 (2) K
0.46 × 0.18 × 0.15 mm

#### Data collection

Oxford Diffraction Gemini R diffractometer
Absorption correction: multi-scan (*CrysAlis RED*; Oxford Diffraction, 2007 ▶) *T*_min_ = 0.945, *T*_max_ = 1.000 (expected range = 0.676–0.716)
27164 measured reflections
8407 independent reflections
4388 reflections with *I* > 2σ(*I*)
*R*_int_ = 0.041

#### Refinement

*R*[*F* ^2^ > 2σ(*F* ^2^)] = 0.047
*wR*(*F* ^2^) = 0.123
*S* = 0.92
8407 reflections
289 parameters
H-atom parameters constrained
Δρ_max_ = 2.03 e Å^−3^
Δρ_min_ = −0.70 e Å^−3^

### 

Data collection: *CrysAlis CCD* (Oxford Diffraction, 2007 ▶); cell refinement: *CrysAlis RED* (Oxford Diffraction, 2007 ▶); data reduction: *CrysAlis RED*; program(s) used to solve structure: *SHELXS97* (Sheldrick, 2008 ▶); program(s) used to refine structure: *SHELXL97* (Sheldrick, 2008 ▶); molecular graphics: *SHELXTL* (Sheldrick, 2008 ▶); software used to prepare material for publication: *SHELXTL*.

## Supplementary Material

Crystal structure: contains datablocks global, I. DOI: 10.1107/S1600536808039214/bq2099sup1.cif

Structure factors: contains datablocks I. DOI: 10.1107/S1600536808039214/bq2099Isup2.hkl

Additional supplementary materials:  crystallographic information; 3D view; checkCIF report

## Figures and Tables

**Table 1:** Hydrogen-bond geometry (Å, °)

*D*—H⋯*A*	*D*—H	H⋯*A*	*D*⋯*A*	*D*—H⋯*A*
C5—H5*A*⋯O1*B*^i^	0.95	2.51	3.209 (3)	131
C4*B*—H4*BA*⋯O1^ii^	0.95	2.67	3.335 (3)	128
C6*B*—H6*BA*⋯O2^iii^	0.95	2.65	3.296 (3)	126

Symmetry codes: (i) 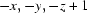; (ii) 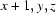; (iii) 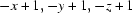.

**Fig. 1.**
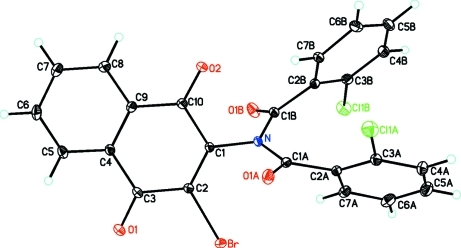
View of (I) showing the atom-labelling scheme. Displacement ellipsoids are drawn at the 20% probability level.

**Fig. 2.**
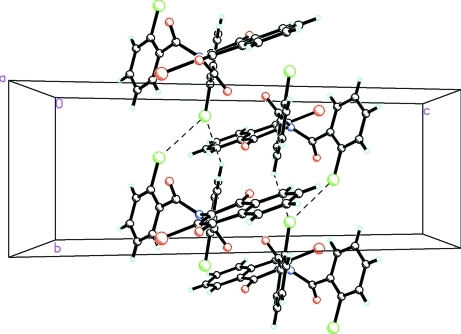
View of the packing viewed down the a axis. Dashed bonds show weak C—H···O interactions and close Cl···Cl contacts.

## supplementary crystallographic information

### Comment

Certain compounds with the chloro-1,4-naphthoquinone skeleton were reported to exhibit antineoplastic property and some have inhibitory effects on human cytomegalovirus (HCMV) protease (Chang *et al.*, 1999; Ertl *et al.*, 1999). The amido and imido derivatives of 3-chloro-1,4-naphthoquinone have been reported to exhibit appreciable anti-inflammatory, antiplatelet, antiallergic and anticancer activities (Lien *et al.*, 1997; Huang *et al.*, 1998; Bakare *et al.*, 2003; Copeland *et al.*,2007) We have developed some imido-substituted 2-chloro-1,4-naphthoquinones with cytotoxic activities on some prostate cancer cell lines. In continuation of our work, the title compound (I) was synthesized as a potential anticancer agent.

Each of the phenyl groups is inclined at about 60° to the naphthoquinone ring of the titled compound C_24_H_12_BrCl_2_NO_4_. The two chlorophenyl rings adopt a conformation that ensures that chlorine substituents are anti to each other so as to reduce electronic repulsion. An examination of the packing shows close contacts between O1A and Br at (1/2 - *x*, 1/2 + *y*, 3/2 - *z*) (2.947 (2) Å) and Cl1A and Cl1B at (*x*, 1 + *y*, *z*) (3.346 (1) Å). The explanation of these close contacts lies in a balance between the torsion angles subtended at the N which balances short intramolecular contacts against short intermolecular contacts and comes up with the best compromise.

### Experimental

To a solution of 2-amino-3-bromo-1,4-naphthoquinone (300 mg, 1.21 mmol) in dry THF was added NaH (72.6 mg 3.025 mmol) and the mixture was stirred for 15 minutes. 2-Chloro-benzoylchloride (0.37 ml) was added thereafter and this mixture was stirred at room temperature for 16–24 hr under argon. The solvent was removed *in vacuo* and the solid residue was dissolved in dichloromethane (40 ml). The resultant solution was washed with water (3 *x* 15 ml), saturated NaCl solution (2 *x* 15 ml) and dried over anhydrous magnesium sulfate. The solvent was removed *in vacuo* and the residue triturated in ethyl acetate to give a yellow solid (280.0 g m). This was recrystallized in ethyl acetate to furnish the title imide (214.2 mg, 34%).

### Refinement

All H atoms were placed in geometrically idealized positions and constrained to ride on their parent atoms with C—H distances of 0.95 Å and *U*_iso_(H) = 1.2*U*_eq_(C).

### Crystal data

**Table:**

C_24_H_12_BrCl_2_NO_4_	*F*(000) = 1056
*M**_r_* = 529.16	*D*_x_ = 1.660 Mg m^−^^3^
Monoclinic, *P*2_1_/*n*	Mo *K*α radiation, λ = 0.71073 Å
*a* = 12.8590 (3) Å	Cell parameters from 6385 reflections
*b* = 7.8126 (1) Å	θ = 4.5–34.8°
*c* = 21.9574 (4) Å	µ = 2.23 mm^−^^1^
β = 106.272 (2)°	*T* = 200 K
*V* = 2117.53 (7) Å^3^	Needle, pale yellow
*Z* = 4	0.46 × 0.18 × 0.15 mm

### Data collection

**Table:**

Oxford Diffraction Gemini R diffractometer	8407 independent reflections
Radiation source: fine-focus sealed tube	4388 reflections with *I* > 2σ(*I*)
graphite	*R*_int_ = 0.041
Detector resolution: 10.5081 pixels mm^-1^	θ_max_ = 34.8°, θ_min_ = 4.5°
φ and ω scans	*h* = −19→20
Absorption correction: multi-scan (*CrysAlis RED*; Oxford Diffraction, 2007)	*k* = −12→12
*T*_min_ = 0.945, *T*_max_ = 1.000	*l* = −34→28
27164 measured reflections	

### Refinement

**Table:**

Refinement on *F*^2^	Primary atom site location: structure-invariant direct methods
Least-squares matrix: full	Secondary atom site location: difference Fourier map
*R*[*F*^2^ > 2σ(*F*^2^)] = 0.047	Hydrogen site location: inferred from neighbouring sites
*wR*(*F*^2^) = 0.123	H-atom parameters constrained
*S* = 0.92	*w* = 1/[σ^2^(*F*_o_^2^) + (0.0618*P*)^2^] where *P* = (*F*_o_^2^ + 2*F*_c_^2^)/3
8407 reflections	(Δ/σ)_max_ = 0.003
289 parameters	Δρ_max_ = 2.03 e Å^−^^3^
0 restraints	Δρ_min_ = −0.70 e Å^−^^3^

### Special details

**Table:**

Geometry. All e.s.d.'s (except the e.s.d. in the dihedral angle between two l.s. planes) are estimated using the full covariance matrix. The cell e.s.d.'s are taken into account individually in the estimation of e.s.d.'s in distances, angles and torsion angles; correlations between e.s.d.'s in cell parameters are only used when they are defined by crystal symmetry. An approximate (isotropic) treatment of cell e.s.d.'s is used for estimating e.s.d.'s involving l.s. planes.
Refinement. Refinement of *F*^2^ against ALL reflections. The weighted *R*-factor *wR* and goodness of fit *S* are based on *F*^2^, conventional *R*-factors *R* are based on *F*, with *F* set to zero for negative *F*^2^. The threshold expression of *F*^2^ > σ(*F*^2^) is used only for calculating *R*-factors(gt) *etc*. and is not relevant to the choice of reflections for refinement. *R*-factors based on *F*^2^ are statistically about twice as large as those based on *F*, and *R*- factors based on ALL data will be even larger.

### Fractional atomic coordinates and isotropic or equivalent isotropic displacement parameters (Å^2^)

**Table:**

	*x*	*y*	*z*	*U*_iso_*/*U*_eq_	
Br	0.16560 (2)	0.07777 (4)	0.693146 (11)	0.03453 (9)	
Cl1A	0.54339 (7)	0.57382 (8)	0.71533 (4)	0.04354 (18)	
Cl1B	0.56864 (7)	−0.14656 (8)	0.60493 (4)	0.04445 (19)	
O1	−0.05313 (17)	0.1335 (4)	0.60398 (10)	0.0601 (7)	
O2	0.24523 (15)	0.3427 (3)	0.49462 (8)	0.0406 (5)	
O1A	0.31064 (15)	0.4218 (2)	0.68149 (8)	0.0325 (4)	
O1B	0.33990 (16)	−0.0028 (2)	0.54700 (9)	0.0389 (5)	
N	0.32004 (15)	0.2100 (2)	0.61270 (8)	0.0207 (4)	
C1	0.20724 (18)	0.2157 (3)	0.58269 (10)	0.0207 (4)	
C2	0.1317 (2)	0.1653 (3)	0.61071 (10)	0.0251 (5)	
C3	0.0139 (2)	0.1748 (3)	0.57732 (11)	0.0298 (5)	
C4	−0.01873 (19)	0.2343 (3)	0.51068 (10)	0.0239 (5)	
C5	−0.1271 (2)	0.2365 (3)	0.47668 (11)	0.0290 (5)	
H5A	−0.1803	0.1963	0.4957	0.035*	
C6	−0.1578 (2)	0.2969 (3)	0.41533 (12)	0.0342 (6)	
H6A	−0.2323	0.2996	0.3925	0.041*	
C7	−0.0814 (2)	0.3536 (4)	0.38673 (12)	0.0367 (6)	
H7A	−0.1037	0.3955	0.3444	0.044*	
C8	0.0275 (2)	0.3495 (3)	0.41937 (11)	0.0333 (6)	
H8A	0.0800	0.3873	0.3994	0.040*	
C9	0.0601 (2)	0.2897 (3)	0.48187 (10)	0.0237 (5)	
C10	0.1758 (2)	0.2874 (3)	0.51699 (10)	0.0252 (5)	
C1A	0.36326 (19)	0.3069 (3)	0.66818 (10)	0.0211 (4)	
C2A	0.47008 (19)	0.2488 (3)	0.71062 (10)	0.0235 (5)	
C3A	0.5541 (2)	0.3614 (3)	0.73579 (11)	0.0292 (5)	
C4A	0.6516 (2)	0.3043 (4)	0.77548 (12)	0.0405 (7)	
H4AA	0.7095	0.3823	0.7914	0.049*	
C5A	0.6642 (3)	0.1328 (4)	0.79186 (13)	0.0454 (7)	
H5AA	0.7309	0.0933	0.8192	0.054*	
C6A	0.5810 (3)	0.0197 (4)	0.76886 (12)	0.0404 (7)	
H6AA	0.5896	−0.0974	0.7809	0.048*	
C7A	0.4848 (2)	0.0758 (3)	0.72825 (11)	0.0298 (5)	
H7AA	0.4277	−0.0035	0.7120	0.036*	
C1B	0.38232 (19)	0.1143 (3)	0.57957 (11)	0.0235 (5)	
C2B	0.49383 (19)	0.1791 (3)	0.58353 (10)	0.0228 (5)	
C3B	0.5818 (2)	0.0694 (3)	0.59230 (11)	0.0260 (5)	
C4B	0.6829 (2)	0.1307 (3)	0.59320 (12)	0.0319 (6)	
H4BA	0.7432	0.0552	0.6007	0.038*	
C5B	0.6956 (2)	0.3040 (3)	0.58300 (12)	0.0323 (6)	
H5BA	0.7648	0.3470	0.5832	0.039*	
C6B	0.6095 (2)	0.4132 (3)	0.57274 (12)	0.0307 (5)	
H6BA	0.6186	0.5308	0.5646	0.037*	
C7B	0.5094 (2)	0.3529 (3)	0.57424 (10)	0.0252 (5)	
H7BA	0.4505	0.4304	0.5689	0.030*	

### Atomic displacement parameters (Å^2^)

**Table:**

	*U*^11^	*U*^22^	*U*^33^	*U*^12^	*U*^13^	*U*^23^
Br	0.02744 (14)	0.05392 (17)	0.02277 (12)	−0.00532 (12)	0.00791 (9)	0.01052 (11)
Cl1A	0.0473 (5)	0.0356 (3)	0.0481 (4)	−0.0096 (3)	0.0140 (3)	−0.0032 (3)
Cl1B	0.0482 (5)	0.0279 (3)	0.0601 (5)	0.0041 (3)	0.0198 (4)	0.0029 (3)
O1	0.0209 (11)	0.120 (2)	0.0407 (12)	−0.0055 (12)	0.0117 (9)	0.0258 (13)
O2	0.0219 (10)	0.0688 (12)	0.0316 (10)	−0.0072 (9)	0.0084 (8)	0.0174 (9)
O1A	0.0269 (10)	0.0382 (9)	0.0299 (9)	0.0062 (8)	0.0040 (7)	−0.0080 (8)
O1B	0.0308 (11)	0.0415 (10)	0.0461 (11)	−0.0133 (9)	0.0134 (8)	−0.0235 (9)
N	0.0144 (9)	0.0301 (9)	0.0175 (8)	−0.0022 (8)	0.0044 (7)	−0.0041 (7)
C1	0.0158 (11)	0.0288 (11)	0.0170 (10)	−0.0020 (9)	0.0040 (8)	−0.0006 (9)
C2	0.0196 (12)	0.0373 (13)	0.0190 (10)	−0.0022 (10)	0.0061 (9)	0.0007 (9)
C3	0.0178 (12)	0.0454 (14)	0.0279 (12)	−0.0019 (11)	0.0090 (10)	0.0050 (11)
C4	0.0178 (12)	0.0308 (12)	0.0222 (11)	−0.0022 (10)	0.0043 (8)	−0.0022 (9)
C5	0.0175 (12)	0.0344 (13)	0.0341 (13)	−0.0033 (10)	0.0056 (10)	−0.0015 (11)
C6	0.0199 (13)	0.0412 (14)	0.0349 (13)	0.0011 (11)	−0.0033 (10)	−0.0042 (12)
C7	0.0324 (16)	0.0460 (15)	0.0258 (12)	0.0015 (13)	−0.0016 (11)	0.0050 (11)
C8	0.0289 (15)	0.0462 (14)	0.0242 (12)	−0.0028 (12)	0.0065 (10)	0.0057 (11)
C9	0.0216 (12)	0.0282 (11)	0.0195 (10)	−0.0017 (10)	0.0028 (8)	0.0004 (9)
C10	0.0209 (12)	0.0329 (12)	0.0217 (10)	−0.0036 (10)	0.0062 (9)	0.0003 (9)
C1A	0.0212 (12)	0.0238 (10)	0.0179 (10)	−0.0029 (9)	0.0047 (8)	0.0008 (8)
C2A	0.0197 (12)	0.0345 (12)	0.0163 (9)	−0.0008 (10)	0.0051 (8)	−0.0021 (9)
C3A	0.0252 (13)	0.0389 (13)	0.0235 (11)	−0.0071 (11)	0.0068 (10)	−0.0034 (10)
C4A	0.0286 (15)	0.0624 (18)	0.0266 (13)	−0.0115 (14)	0.0009 (11)	−0.0042 (13)
C5A	0.0344 (17)	0.0663 (19)	0.0318 (14)	0.0121 (15)	0.0031 (12)	0.0050 (14)
C6A	0.049 (2)	0.0436 (15)	0.0270 (13)	0.0128 (14)	0.0079 (12)	0.0035 (12)
C7A	0.0329 (14)	0.0333 (12)	0.0222 (11)	0.0048 (11)	0.0058 (9)	−0.0010 (10)
C1B	0.0189 (12)	0.0303 (12)	0.0221 (10)	0.0003 (9)	0.0070 (9)	−0.0014 (9)
C2B	0.0200 (12)	0.0318 (12)	0.0165 (9)	0.0000 (10)	0.0051 (8)	−0.0029 (9)
C3B	0.0265 (13)	0.0287 (11)	0.0251 (11)	0.0048 (11)	0.0111 (9)	0.0001 (10)
C4B	0.0249 (14)	0.0430 (14)	0.0290 (12)	0.0044 (11)	0.0094 (10)	0.0000 (11)
C5B	0.0241 (14)	0.0441 (14)	0.0313 (13)	−0.0098 (12)	0.0119 (10)	−0.0066 (11)
C6B	0.0326 (14)	0.0322 (12)	0.0309 (12)	−0.0063 (12)	0.0148 (10)	−0.0015 (11)
C7B	0.0201 (12)	0.0349 (12)	0.0221 (11)	−0.0026 (10)	0.0083 (9)	−0.0004 (10)

### Geometric parameters (Å, °)

**Table:**

Br—C2	1.869 (2)	C1A—C2A	1.498 (3)
Cl1A—C3A	1.715 (3)	C2A—C3A	1.383 (3)
Cl1B—C3B	1.726 (2)	C2A—C7A	1.404 (3)
O1—C3	1.214 (3)	C3A—C4A	1.384 (4)
O2—C10	1.213 (3)	C4A—C5A	1.385 (4)
O1A—C1A	1.209 (3)	C4A—H4AA	0.9500
O1B—C1B	1.195 (3)	C5A—C6A	1.370 (5)
N—C1A	1.410 (3)	C5A—H5AA	0.9500
N—C1	1.416 (3)	C6A—C7A	1.378 (4)
N—C1B	1.434 (3)	C6A—H6AA	0.9500
C1—C2	1.346 (3)	C7A—H7AA	0.9500
C1—C10	1.494 (3)	C1B—C2B	1.500 (3)
C2—C3	1.489 (3)	C2B—C3B	1.389 (3)
C3—C4	1.479 (3)	C2B—C7B	1.396 (3)
C4—C5	1.384 (3)	C3B—C4B	1.381 (4)
C4—C9	1.405 (3)	C4B—C5B	1.390 (4)
C5—C6	1.377 (3)	C4B—H4BA	0.9500
C5—H5A	0.9500	C5B—C6B	1.365 (4)
C6—C7	1.378 (4)	C5B—H5BA	0.9500
C6—H6A	0.9500	C6B—C7B	1.380 (4)
C7—C8	1.383 (4)	C6B—H6BA	0.9500
C7—H7A	0.9500	C7B—H7BA	0.9500
C8—C9	1.398 (3)	H6AA—Cl1A^i^	2.923
C8—H8A	0.9500	H6BA—Cl1B^ii^	2.805
C9—C10	1.471 (3)		
			
C1A—N—C1	119.32 (18)	C7A—C2A—C1A	119.5 (2)
C1A—N—C1B	125.28 (19)	C2A—C3A—C4A	121.0 (3)
C1—N—C1B	115.17 (17)	C2A—C3A—Cl1A	120.9 (2)
C2—C1—N	123.7 (2)	C4A—C3A—Cl1A	118.0 (2)
C2—C1—C10	121.1 (2)	C3A—C4A—C5A	119.6 (3)
N—C1—C10	115.22 (18)	C3A—C4A—H4AA	120.2
C1—C2—C3	121.7 (2)	C5A—C4A—H4AA	120.2
C1—C2—Br	123.22 (18)	C6A—C5A—C4A	120.4 (3)
C3—C2—Br	115.11 (16)	C6A—C5A—H5AA	119.8
O1—C3—C4	121.2 (2)	C4A—C5A—H5AA	119.8
O1—C3—C2	120.8 (2)	C5A—C6A—C7A	120.0 (3)
C4—C3—C2	118.1 (2)	C5A—C6A—H6AA	120.0
C5—C4—C9	119.8 (2)	C7A—C6A—H6AA	120.0
C5—C4—C3	120.1 (2)	C6A—C7A—C2A	120.8 (3)
C9—C4—C3	120.1 (2)	C6A—C7A—H7AA	119.6
C6—C5—C4	120.1 (2)	C2A—C7A—H7AA	119.6
C6—C5—H5A	120.0	O1B—C1B—N	118.5 (2)
C4—C5—H5A	120.0	O1B—C1B—C2B	124.2 (2)
C5—C6—C7	120.7 (2)	N—C1B—C2B	117.08 (19)
C5—C6—H6A	119.7	C3B—C2B—C7B	118.4 (2)
C7—C6—H6A	119.7	C3B—C2B—C1B	121.8 (2)
C6—C7—C8	120.2 (2)	C7B—C2B—C1B	119.6 (2)
C6—C7—H7A	119.9	C4B—C3B—C2B	121.0 (2)
C8—C7—H7A	119.9	C4B—C3B—Cl1B	118.11 (19)
C7—C8—C9	119.9 (2)	C2B—C3B—Cl1B	120.86 (19)
C7—C8—H8A	120.1	C3B—C4B—C5B	119.2 (2)
C9—C8—H8A	120.1	C3B—C4B—H4BA	120.4
C8—C9—C4	119.3 (2)	C5B—C4B—H4BA	120.4
C8—C9—C10	120.0 (2)	C6B—C5B—C4B	120.6 (2)
C4—C9—C10	120.7 (2)	C6B—C5B—H5BA	119.7
O2—C10—C9	122.3 (2)	C4B—C5B—H5BA	119.7
O2—C10—C1	119.6 (2)	C5B—C6B—C7B	120.1 (2)
C9—C10—C1	118.1 (2)	C5B—C6B—H6BA	119.9
O1A—C1A—N	119.8 (2)	C7B—C6B—H6BA	119.9
O1A—C1A—C2A	123.5 (2)	C6B—C7B—C2B	120.5 (2)
N—C1A—C2A	116.47 (19)	C6B—C7B—H7BA	119.7
C3A—C2A—C7A	118.2 (2)	C2B—C7B—H7BA	119.7
C3A—C2A—C1A	122.3 (2)		
			
C1A—N—C1—C2	−61.2 (3)	C1—N—C1A—C2A	157.69 (19)
C1B—N—C1—C2	124.0 (2)	C1B—N—C1A—C2A	−28.1 (3)
C1A—N—C1—C10	117.1 (2)	O1A—C1A—C2A—C3A	−51.8 (3)
C1B—N—C1—C10	−57.7 (3)	N—C1A—C2A—C3A	133.8 (2)
N—C1—C2—C3	179.6 (2)	O1A—C1A—C2A—C7A	125.2 (2)
C10—C1—C2—C3	1.4 (4)	N—C1A—C2A—C7A	−49.3 (3)
N—C1—C2—Br	−0.8 (3)	C7A—C2A—C3A—C4A	2.3 (4)
C10—C1—C2—Br	−179.02 (17)	C1A—C2A—C3A—C4A	179.3 (2)
C1—C2—C3—O1	−177.8 (3)	C7A—C2A—C3A—Cl1A	178.70 (18)
Br—C2—C3—O1	2.5 (4)	C1A—C2A—C3A—Cl1A	−4.3 (3)
C1—C2—C3—C4	2.8 (4)	C2A—C3A—C4A—C5A	−2.0 (4)
Br—C2—C3—C4	−176.85 (17)	Cl1A—C3A—C4A—C5A	−178.5 (2)
O1—C3—C4—C5	−2.9 (4)	C3A—C4A—C5A—C6A	0.2 (4)
C2—C3—C4—C5	176.5 (2)	C4A—C5A—C6A—C7A	1.2 (4)
O1—C3—C4—C9	176.8 (3)	C5A—C6A—C7A—C2A	−0.9 (4)
C2—C3—C4—C9	−3.9 (4)	C3A—C2A—C7A—C6A	−0.9 (4)
C9—C4—C5—C6	−1.7 (4)	C1A—C2A—C7A—C6A	−177.9 (2)
C3—C4—C5—C6	177.9 (2)	C1A—N—C1B—O1B	156.2 (2)
C4—C5—C6—C7	0.9 (4)	C1—N—C1B—O1B	−29.3 (3)
C5—C6—C7—C8	0.3 (4)	C1A—N—C1B—C2B	−29.4 (3)
C6—C7—C8—C9	−0.8 (4)	C1—N—C1B—C2B	145.08 (19)
C7—C8—C9—C4	0.0 (4)	O1B—C1B—C2B—C3B	−48.2 (3)
C7—C8—C9—C10	−179.2 (2)	N—C1B—C2B—C3B	137.8 (2)
C5—C4—C9—C8	1.2 (4)	O1B—C1B—C2B—C7B	127.7 (3)
C3—C4—C9—C8	−178.4 (2)	N—C1B—C2B—C7B	−46.3 (3)
C5—C4—C9—C10	−179.6 (2)	C7B—C2B—C3B—C4B	1.4 (3)
C3—C4—C9—C10	0.8 (3)	C1B—C2B—C3B—C4B	177.3 (2)
C8—C9—C10—O2	3.2 (4)	C7B—C2B—C3B—Cl1B	179.26 (17)
C4—C9—C10—O2	−176.0 (2)	C1B—C2B—C3B—Cl1B	−4.8 (3)
C8—C9—C10—C1	−177.5 (2)	C2B—C3B—C4B—C5B	−2.2 (3)
C4—C9—C10—C1	3.3 (3)	Cl1B—C3B—C4B—C5B	179.85 (19)
C2—C1—C10—O2	174.9 (2)	C3B—C4B—C5B—C6B	0.6 (4)
N—C1—C10—O2	−3.4 (3)	C4B—C5B—C6B—C7B	1.9 (4)
C2—C1—C10—C9	−4.4 (3)	C5B—C6B—C7B—C2B	−2.7 (4)
N—C1—C10—C9	177.21 (19)	C3B—C2B—C7B—C6B	1.1 (3)
C1—N—C1A—O1A	−17.0 (3)	C1B—C2B—C7B—C6B	−174.9 (2)
C1B—N—C1A—O1A	157.3 (2)		

Symmetry codes: (i) *x*, *y*−1, *z*; (ii) *x*, *y*+1, *z*.

### Hydrogen-bond geometry (Å, °)

**Table:**

*D*—H···*A*	*D*—H	H···*A*	*D*···*A*	*D*—H···*A*
C5—H5A···O1B^iii^	0.95	2.51	3.209 (3)	131
C4B—H4BA···O1^iv^	0.95	2.67	3.335 (3)	128
C6B—H6BA···O2^v^	0.95	2.65	3.296 (3)	126

Symmetry codes: (iii) −*x*, −*y*, −*z*+1; (iv) *x*+1, *y*, *z*; (v) −*x*+1, −*y*+1, −*z*+1.
